# Anti-fibrotic effect of human amniotic fluid stem cells in biliary epithelial-mesenchymal transition of liver ductal organoid

**DOI:** 10.1093/stcltm/szaf052

**Published:** 2025-11-03

**Authors:** Sinobol Chusilp, Poramate Klanrit, Carol Lee, Dorothy Lee, Bo Li, Felicia Balsamo, Kanokrat Thaiwatcharamas, Patchareeporn Tanming, Dolrudee Aroonsaeng, Paisarn Vejchapipat, Agostino Pierro

**Affiliations:** Division of Pediatric Surgery, Department of Surgery, Faculty of Medicine, Khon Kaen University, Khon Kaen, 40002, Thailand; Division of General and Thoracic Surgery, Translational Medicine Program, The Hospital for Sick Children, Toronto, ON, M5G 1X8, Canada; Department of Systems Biosciences and Computational Medicine, Faculty of Medicine, Khon Kaen University, Khon Kaen, 40002, Thailand; Division of General and Thoracic Surgery, Translational Medicine Program, The Hospital for Sick Children, Toronto, ON, M5G 1X8, Canada; Division of General and Thoracic Surgery, Translational Medicine Program, The Hospital for Sick Children, Toronto, ON, M5G 1X8, Canada; Division of General and Thoracic Surgery, Translational Medicine Program, The Hospital for Sick Children, Toronto, ON, M5G 1X8, Canada; Division of General and Thoracic Surgery, Translational Medicine Program, The Hospital for Sick Children, Toronto, ON, M5G 1X8, Canada; Division of Pediatric Surgery, Department of Surgery, Faculty of Medicine, Khon Kaen University, Khon Kaen, 40002, Thailand; Division of Pediatric Surgery, Department of Surgery, Faculty of Medicine, Khon Kaen University, Khon Kaen, 40002, Thailand; Division of Pediatric Surgery, Department of Surgery, Faculty of Medicine, Khon Kaen University, Khon Kaen, 40002, Thailand; Division of Pediatric Surgery, Department of Surgery, Faculty of Medicine, Chulalongkorn University, Bangkok, 10330, Thailand; Division of General and Thoracic Surgery, Translational Medicine Program, The Hospital for Sick Children, Toronto, ON, M5G 1X8, Canada

**Keywords:** amniotic fluid stem cell, bile duct, epithelial-mesenchymal transition, organoids

## Abstract

**Purpose:**

In biliary atresia (BA), it has been demonstrated that biliary epithelial-mesenchymal transition (EMT) of reactive ductular cells is associated with liver fibrosis. This study aimed to develop an *ex vivo* biliary EMT model of liver ductal organoids for exploring the involvement of biliary EMT in fibrogenesis and to investigate whether human amniotic fluid stem cells (hAFSCs) can mitigate the biliary EMT process.

**Methods:**

Liver ductal organoids were generated from the intrahepatic bile duct of healthy neonatal mice. Biliary EMT was induced in organoids by the administration of transforming growth factor beta-1 (TGF-β1) in culture medium. hAFSCs were co-cultured with organoids during biliary EMT induction. Expression of biliary epithelial cells, mesenchymal cells, myofibroblast, collagen I, and genes related to the Wnt signaling pathway were evaluated.

**Results:**

Following administration of TGF-β1, we observed an increased expression of mesenchymal cell markers N-cadherin and Vimentin, as well as myofibroblast marker alpha-smooth muscle actin (α-SMA) in liver ductal organoids which were associated with increased expression of collagen 1. Administration of hAFSCs to organoids significantly attenuated TGF-β1-induced biliary EMT and collagen production. In addition, Wnt signaling was upregulated in biliary EMT, while hAFSCs downregulated the Wnt signaling resulting in decreased expression of myofibroblast and collagen in organoids.

**Conclusion:**

TGF-β1 is a potent cytokine that induces biliary EMT. hAFSCs significantly mitigated TGF-β1-induced biliary EMT in liver ductal organoids. The beneficial effect of hAFSCs administration is associated with the downregulation of the Wnt signaling pathway. This study indicates that hAFSCs can prevent the progression of liver fibrosis in BA.

Significance statement Biliary atresia (BA) causes progressive fibrosis leading to pediatric liver failure. Using a biliary organoid model, we show that TGF-β1 drives epithelial–mesenchymal transition (EMT) and collagen deposition, while human amniotic fluid stem cells (hAFSCs) block this process via Wnt pathway suppression. These findings highlight hAFSCs as a potential anti-fibrotic therapy for BA.

## Introduction

Biliary atresia (BA) is a progressive inflammatory disease of both intra- and extrahepatic bile ducts occurring in infants, which leads to fibro-obliteration of bile ducts and progression of liver fibrosis.^1^^,^^2^ Although the Kasai portoenterostomy^3^ has greatly improved the survival with native liver, BA patients who underwent this operation still suffer from liver-related complications due to the progression of liver fibrosis to cirrhosis and ultimately require liver transplantation.^4–8^ Liver fibrosis is a healing process resulting from chronic liver injury. It is characterized by excessive accumulation of extracellular matrix components including collagen in the liver. In BA, liver fibrosis progresses even if bile flow is successfully restored by Kasai portoenterostomy indicating a complicated pathophysiology other than just a consequence of bile duct obstruction.^9^^,^^10^

Reactive ductular cells (RDCs) or proliferating bile ducts are present in the liver during BA and are related to the degree of liver fibrosis and long-term native liver survival.^11–15^ However, the mechanism of RDCs in driving liver fibrosis is not fully understood. Several studies revealed that RDCs in BA liver express both biliary epithelial cell markers and myofibroblast markers suggesting the occurrence of a biliary epithelial-mesenchymal transition (EMT) process in RDCs.^11–18^ In this process, the reactive biliary phenotype of RDCs is transforming into myofibroblast phenotype which gains the ability to produce extracellular matrix components and results in fibrogenesis.^19^^,^^20^ Thus, understanding the pathways involved in biliary EMT process is crucial for identifying potential targets and developing of anti-fibrotic therapy that can halt biliary EMT of RDCs and decelerate the progression of liver fibrosis in BA.

The Wnt signaling pathway is a crucial pathway involved in liver development, homeostasis, and regeneration. Aberrant activation of the Wnt signaling pathway occurs in various liver pathologies including liver fibrosis. In addition, modulation of Wnt molecules has been shown to reduce fibrogenesis.^21^^,^^22^ In biliary epithelial cells, it has been reported that Wnt signaling involves the development of biliary fibrosis and promotes RDCs proliferation and differentiation after bile duct injury.^23–25^ However, the role of the Wnt signaling pathway in biliary EMT process of RDCs has not been characterized.

Transforming growth factor-β (TGF-β) is a cytokine that plays an important role in fibrogenesis^26^ and is a strong inducer of EMT.^27^ Analysis of BA liver demonstrated that TGF-β1 is expressed in both hepatocyte and biliary epithelial cells,^28^ and an increase of TGF-β1 expression level in BA liver is significantly correlated with liver fibrosis.^28^^,^^29^ However, the effect of TGF-β1 in inducing biliary EMT of RDCs in BA remains unclear.

Liver ductal organoids are mini-organ structures derived from resident progenitor cells of intrahepatic bile ducts.^30^ They comprise cholangiocytes, which harbor liver progenitor cells.^31^^,^^32^ To generate liver ductal organoids, bile duct fragments are isolated from the liver, cultured in Matrigel domes as extracellular matrix, and supplemented with optimized growth factors in the culture medium.^30^^,^^33^ The organoids are then self-organized into single-layer epithelial spheroids with a lumen on the inside, mimicking *in vivo* intrahepatic bile ducts. Moreover, they are capable of self-renewal. Therefore, these organoids are suitable for modeling diseases related to bile duct such as BA.

Amniotic fluid stem cells (AFSCs) are highly proliferative stem cells in amniotic fluid which can be derived from the developing fetus. These stem cells can differentiate into various cell types with low immunogenicity and lack of tumorigenicity.^34–37^ Furthermore, they can be collected, expanded, and maintained indefinitely for subsequent application in the perinatal period.^37–39^ Various experimental studies have demonstrated that AFSCs have the ability to repair damaged tissue through multiple mechanisms including modulating the immune system, reducing inflammation, inhibiting cell apoptosis, and stimulating tissue regeneration.^40–49^ In addition, it has been reported that AFSCs possess anti-fibrotic potential in several experimental disease models.^50–55^ The mechanism of AFSCs in reducing tissue fibrogenesis is possibly due to the modulation of myofibroblast expression in damaged tissue.^50^^,^^52^

The beneficial effects of human amniotic fluid stem cells (hAFSCs) were previously studied in mouse model of several diseases such as a cutaneous wound model,^50^ an acute tubular necrosis model^56^ and hypoxic-ischemic brain model.^57^ They demonstrated that after transplanting hAFSCs into mice, hAFSCs could engraft in the areas of injury and alleviate organ damage. In addition, we have demonstrated that hAFSCs attenuated cholangiocyte apoptosis in a bile duct injury model of mouse liver ductal organoids.^40^ Therefore, we hypothesized that AFSCs modulate the myofibroblast expression by mitigating the transformation of RDCs into myofibroblasts in the biliary EMT process. Hence, this study aimed (1) to establish an *ex vivo* TGF-β1-induced biliary EMT model of liver ductal organoids for exploring the involvement of biliary EMT in fibrogenesis and (2) to investigate the anti-fibrotic property of hAFSCs in mitigating biliary EMT.

## Material and methods

### Liver ductal organoid culture

Following protocol from StemCell Technologies, liver ductal organoids were generated from intrahepatic bile ducts of healthy post-natal day 9 C57BL/C mouse pups. All animal experiments were approved by the Animal Care Committee at The Hospital for Sick Children (AUP #1000057313). Liver tissue was collected and cut into small pieces (3-5 mm). The intrahepatic bile duct fragments were isolated by digestion using a tissue dissociation cocktail (StemCell Technologies, Cambridge, MA, United States) composed of collagenase IV, dispase, and Dulbecco’s Modified Eagle’s medium/nutrient mixture F-12 (DMEM/F-12) with 15 mM HEPES. During liver digestion, the supernatant containing small fragments of the intrahepatic bile duct was collected, and the intrahepatic bile ducts sized 37-70 μm were isolated from the supernatant by using cell strainers. Then these minute bile ducts that harbored ductal progenitor cells^31^^,^^33^ were cultured in Matrigel domes (Corning, NY, United States), supplemented with mouse HepatiCult organoid growth medium (StemCell Technologies, Cambridge, MA, United States), and maintained in an incubator at 37 °C and 5% CO_2_. The culture medium was changed every 2 days. After 4 days of culture, the organoids were expanded by passaging with a seeding density of 300 fragments per well. The second and third passaged liver ductal organoids were used in this study.

### Ex vivo biliary EMT model using mouse liver ductal organoid

**Figure 1.**
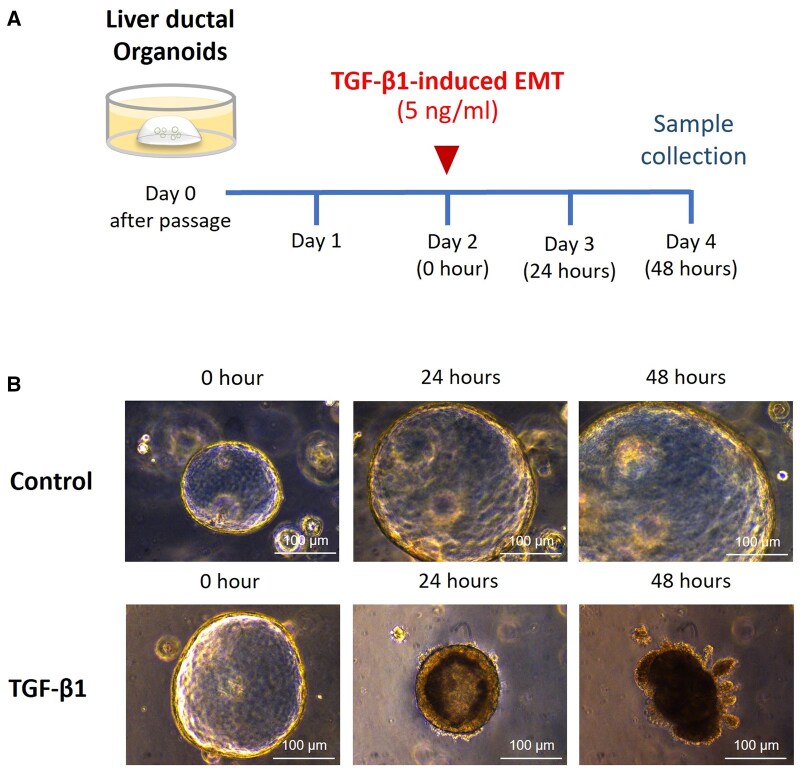
An ex vivo biliary epithelial-mesenchymal transition (EMT) model of mouse liver ductal organoid. (A) Biliary EMT was induced in liver ductal organoids by administration of recombinant mouse TGF-β1 protein (5 ng/mL) in culture medium on day 2 after passage. Organoids were treated with TGF-β1 for 48 hours before harvested for analysis. (B) Morphology of liver ductal organoids after exposure to TGF-β1 at 0 hours, 24 hours, and 48 hours.

To induce biliary EMT in liver ductal organoids, recombinant mouse TGF-β1 protein (5 ng/mL) (R&D systems, Minneapolis, MN, United States) was administrated in culture medium on day 2 after passage of organoids and was maintained for 48 hours. Liver ductal organoids were then harvested for further analysis on day 4 of culture (Figure 1A).

### Human amniotic fluid stem cells (hAFSCs) culture

hAFSCs were purchased from Celprogen (Celprogen, Torrance, CA) and maintained in Human amniotic fluid expansion media with serum (Celprogen, Torrance, CA) at 37 °C and 5% CO_2_. The culture medium was replaced every 3-4 days. For use in experiments, hASFCs were collected after reaching 80%-100% cell confluency by trypsinization and washed using phosphate-buffered saline 3 times.

### Direct and indirect co-culture of hAFSCs and liver ductal organoids

To assess the effect of hAFSCs in an *ex vivo* biliary EMT model of mouse liver ductal organoids, our published methods of direct and indirect co-culture systems were used in this study.^40^ To direct co-culture hAFSCs with liver ductal organoids, 500 cells of hAFSCs together with 300 fragments of organoids were cultured together in Matrigel for 2 days followed by induction of biliary EMT. To create the indirect co-culture system, a transwell membrane insert (Corning, NY, United States) was used in this study. hAFSCs were seeded on a transwell membrane insert at a density of 10 000 cells. The insert was then placed above the Matrigel dome containing organoids of 300 fragments during biliary EMT induction without direct contact with the Matrigel dome.

### Real-time quantitative reverse transcription polymerase chain reaction (RT-qPCR)

Messenger RNA (mRNA) expressions in liver ductal organoids were analyzed using two-step RT-qPCR. Trizol reagent (Invitrogen, Carlsbad, CA, United States) was used for extracting mRNA from the organoids. Complementary DNA (cDNA) was created by using qScript cDNA SuperMix (Quantabio, Beverly, MA, United States) and S1000 Thermal Cycler (Bio-Rad Laboratories, Hercules, CA, United States). RT-qPCR was performed using advanced qPCR Master Mix and CFX384 Real-Time System (Bio-Rad Laboratories, Hercules, CA, United States). *Gapdh* was used as a housekeeping gene for the normalization of gene expression. The sequences of the primers for each gene are shown in Table 1.

**Table 1.** The sequences of the primers.

Gene target	Primer sequence (5′→3′)
*Cytokeratin-19* (Mouse)	Forward: GACGTGCGTGCCGACATAGA Reverse: GGTGGGCAGATTGTTGTAGTGG
*E-cadherin* (Mouse)	Forward: AAAAGAAGGCTGTCCTTGGC Reverse: GAGGTCTACACCTTCCCGGT
*Alpha-fetoprotein* (Mouse)	Forward: TTCGTATTCCAACAGGAGGCTAT Reverse: GTTCAGGCTTTTGCTTCACCA
*Cytokeratin-7* (Mouse)	Forward: TTCCCC GAATCTTTGAGGCT Reverse: TCTTCCACCACATCCTGCAT
*N-cadherin* (Mouse)	Forward: CAGGGTGGACGTCATTGTAG Reverse: AGGGTCTCCACCACTGATTC
*Vimentin* (Mouse)	Forward: AAGGAAGAGATGGCTCGTCA Reverse: TTGAGTGGGTGTCAACCAGA
*α-SMA* (Mouse)	Forward: GGGAGTAATGGTTGGAATGG Reverse: GGTGATGATGCCGTGTTCTA
*Col1a1* (Mouse)	Forward: TGACTGGAAGAGCGGAGAGT Reverse: GACGGCTGAGTAGGGAACAC
*Lef1* (Mouse)	Forward: TACAACAAGGGACCCTCCTAC Reverse: GGAGAAAGGGACCCATTTGAC
*Tcf4* (Mouse)	Forward: CAATCCAGGAACCCTTTCG Reverse: AGGAGCGTAGACTGAAGAC
*Wnt5a* (Mouse)	Forward: CAAATAGGCAGCCGAGAGAC Reverse: CTCTAGCGTCCACGAACTCC
*Wnt7b* (Mouse)	Forward: TTCTGGAGGACCGCATGAA Reverse: GGTCCAGCAAGTTTTGGTGGTA
*Gapdh*	Forward: TGAAGCAGGCATCTGAGGG Reverse: CGAAGGTGGAAGAGTGGGAG

### Immunofluorescent staining

After fixing with 4% paraformaldehyde, liver ductal organoids were washed with cold phosphate-buffered saline to dissolve the Matrigel. Then they were permeabilized using 1% Triton X-100 in phosphate-buffered saline for 1 hour at room temperature and blocked with 5% bovine serum albumin and 1% Triton X-100 in phosphate-buffered saline overnight at 4 °C. Primary antibodies were diluted in 5% bovine serum albumin and 1% Triton X-100 in phosphate-buffered saline. The organoids were incubated with primary antibody for 24 hours at 4 °C followed by washing with 1% Triton X-100 in phosphate-buffered saline 2 times. Alexa Fluor-conjugated secondary antibody (1:300) (Invitrogen, Carlsbad, CA) together with DAPI (1:300) (Vector Laboratories, Newark, CA) were diluted in 5% bovine serum albumin and 1% Triton X-100 in phosphate-buffered saline. The organoids were incubated with secondary antibodies and DAPI overnight at 4 °C and washed with phosphate-buffered saline before imaging by Leica SP8 lightning confocal microscopy (Leica Microsystems, Wetzlar, Germany). The concentrations of primary antibodies are listed in Table 2.

**Table 2.** The concentrations of primary antibodies.

Primary antibodies	Host	Dilution	Company
E-cadherin	Rabbit	1:100	Cell Signaling Technology, MA, United States
N-cadherin	Mouse	1:100	Abcam, Cambridge, United Kingdom
α-SMA	Rabbit	1:100	Abcam, Cambridge, United Kingdom
Collagen 1	Mouse	1:100	Invitrogen, Carlsbad, CA, United States

### Statistical analysis

GraphPad Prism 10 was used for statistical analysis. D’Agostino-Pearson test was used to assess the normality of data. All data was normally distributed and reported as means ± SD. Unpaired *t*-test and one-way ANOVA with Tukey post-hoc test were used for data comparison. Differences were considered statistically significant when *P* < 0.05.

### Data availability

All data are incorporated into the article.

## Results

### An ex vivo biliary EMT model of liver ductal organoids

**Figure 2.**
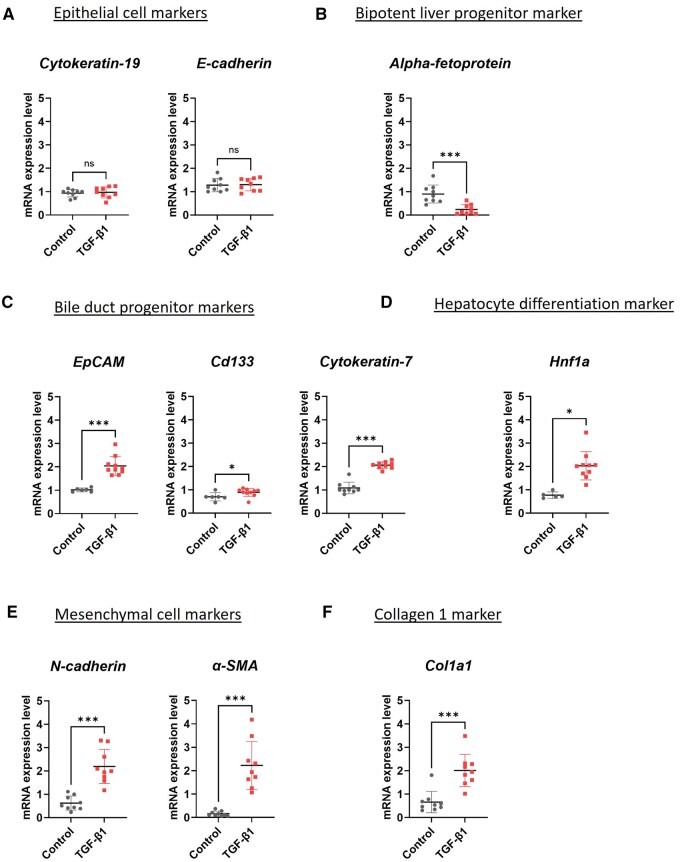
mRNA Expression of genes related to epithelial cell markers, bipotent liver progenitor marker, bile duct progenitor markers, hepatocyte differentiation marker, mesenchymal cell markers, and collagen marker in liver ductal organoids at 48 hours after receiving TGF-β1. (A) mRNA expressions of epithelial cell markers *Cytokeratin-19* and *E-cadherin*. (B) mRNA expressions of bipotent liver progenitor cell marker *Alpha-fetoprotein*. (C) mRNA expressions of liver progenitor cell markers *EpCAM*, *Cd133*, and *Cytokeratin-7*. (D) mRNA expressions of hepatocyte differentiation marker *Hnf1a*. (E) mRNA expressions of mesenchymal cell markers *N-cadherin* and *α-SMA*. (F) mRNA expressions of collagen marker *Col1a1*. The experiments were performed with 3 replicates (total *N* = 9 per group). Data are presented as mean ± SD. ***P < 0.001.

After treating liver ductal organoids with TGF-β1 for 48 hours (Figure 1A), we found that the morphology of organoids gradually changed from a single-layer epithelial spheroids with lumen on the inside into an irregular shape with outgrowth of epithelial cells (Figure 1B). Analysis of mRNA expressions in liver ductal organoids treated with TGF-β1 for 48 hours showed that there were no changes in the expression of biliary epithelial cell markers including *Cytokeratin-19* and *E-cadherin* compared to control organoids (Figure 2A). However, the assessment of liver progenitor cell markers revealed that the organoids treated with TGF-β1 had decreased expression of bipotent liver progenitor cell marker *Alpha-fetoprotein* (Figure 2B), and increased expression of bile duct progenitor markers *EpCAM*, *Cd133*, and *Cytokeratin-7* (Figure 2C), as well as hepatocyte differentiation marker *Hnf1a* (Figure 2D) when compared to control organoids. These results suggest that after exposure to TGF-β1, the organoids differentiate from bipotent liver progenitor into bile duct progenitor and hepatocyte phenotypes.

**Figure 3.**
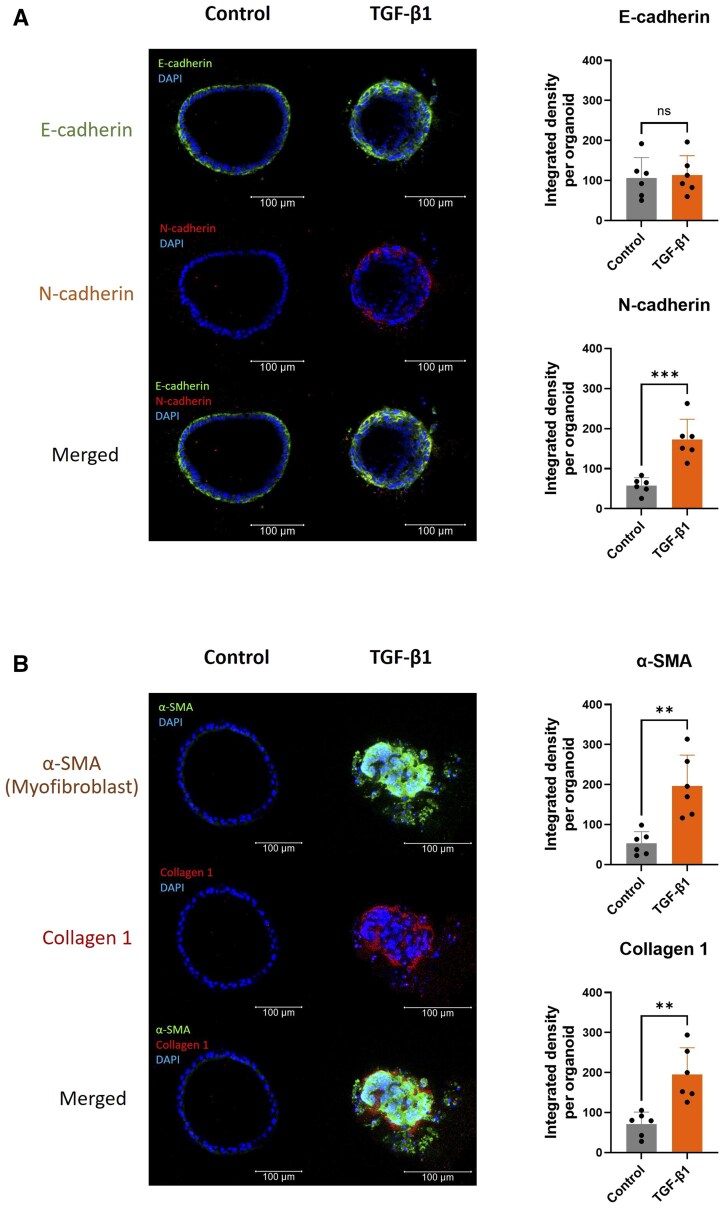
Protein expressions of biliary epithelial cell, mesenchymal cell, myofibroblast, and collagen markers in liver ductal organoids undergoing biliary epithelial-mesenchymal transition (EMT). (A) Representative images of liver ductal organoids in double immunofluorescent staining of epithelial cell marker E-cadherin (green) and mesenchymal cell marker N-cadherin (red), and the quantification of immunofluorescent expression. (B) Representative images of liver ductal organoids in double immunofluorescent staining of myofibroblast marker α-SMA (green) and Collagen 1 (red), and the quantification of immunofluorescent expression. Nuclei were stained with DAPI (blue). Data are presented as mean ± SD. **P < 0.01, ***P < 0.001 (*N* = 6 per group).

When the mRNA expression of mesenchymal cell markers was analyzed, we found that administration of TGF-β1significantly increased the expression of *N-cadherin* and myofibroblast marker *α-smooth muscle actin* (*α-SMA*) (Figure 2E) and increased the expression of collagen marker *Cola1* (Figure 2F). The protein expression of liver ductal organoids was analyzed by immunofluorescence staining. Double immunostaining of epithelial cell marker E-cadherin and mesenchymal cell marker N-cadherin confirmed that there was an increased expression of mesenchymal cells in liver ductal organoids treated with TGF-β1 (Figure 3A). In addition, double staining of liver ductal organoids with myofibroblast marker α-SMA and Collagen1 showed that an increased expression of myofibroblast marker was associated with an increase in collagen expression in the organoids treated with TGF-β1. This co-localization indicates that the myofibroblasts deposit Collagen 1 (Figure 3B). These results indicate that TGF-β1 is a potent inducer of biliary EMT in liver ductal organoids, and this EMT process is associated with an increased collagen expression.

### hAFSCs mitigated biliary EMT in liver ductal organoids in both direct and indirect co-culture systems

To explore the anti-fibrotic potential of hAFSCs in liver ductal organoids, hAFSCs were co-cultured with liver ductal organoids in direct and indirect co-culture systems during biliary EMT induction. The direct co-culture system has been designed to mimic stem cell transplantation by allowing direct contact between hAFSCs and organoids. The indirect co-culture system aims to evaluate the paracrine effect of hAFSCs by using a transwell membrane insert which allows the mediators secreted by hAFSCs to pass through the membrane and interact with the organoids without direct cell contact. The effect of hAFSCs on liver ductal organoids in non-EMT condition was evaluated. We found that without administration of TGF-β1, hAFSCs had no effect on the expressions of epithelial cell, progenitor cell, mesenchymal cell markers, and collagen in the organoids (Figure S1).

**Figure 4.**
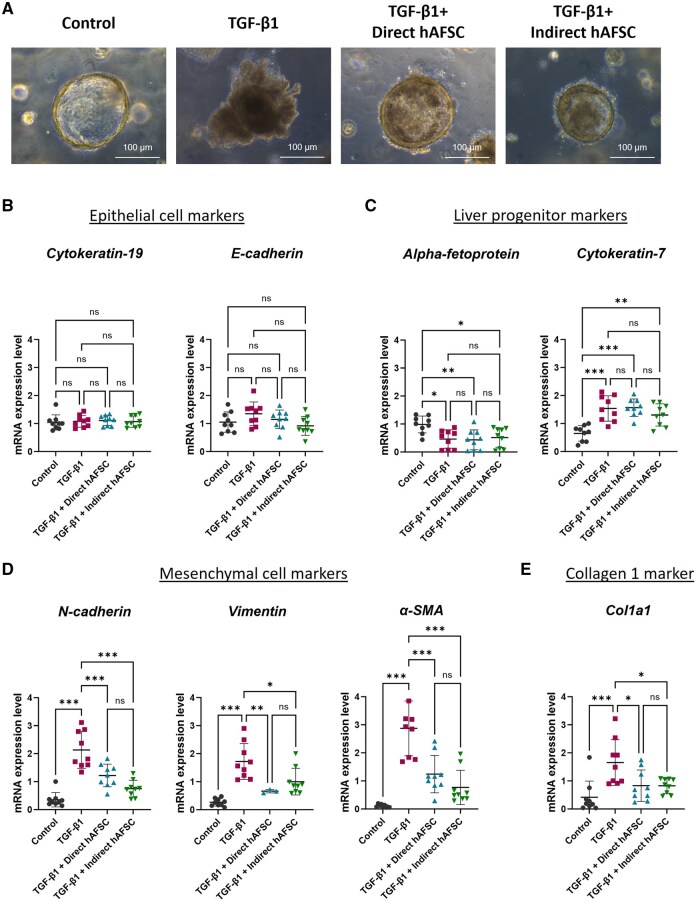
Effect of human amniotic fluid stem cells (hAFSCs) treatment in both direct and indirect co-culture systems in liver ductal organoids undergoing biliary epithelial-mesenchymal transition (EMT). (A) Morphology of liver ductal organoids in control group, TGF-β1 group, TGF-β1 with direct hAFSCs treatment group, and TGF-β1 with indirect hAFSCs treatment group. (B) mRNA expressions of epithelial cell markers *Cytokeratin-19* and *E-cadherin*. (C) mRNA expressions of liver progenitor cell markers *Alpha-fetoprotein* and *Cytokeratin-7*. (D) mRNA expressions of mesenchymal cell markers *N-cadherin*, *Vimentin*, and *α-SMA*. (E) mRNA expressions of collagen marker *Col1a1*. The experiments were performed with 3 replicates (total *N* = 9 per group). Data are presented as mean ± SD. * *P* < 0.05, **P < 0.01, ***P < 0.001.

**Figure 5.**
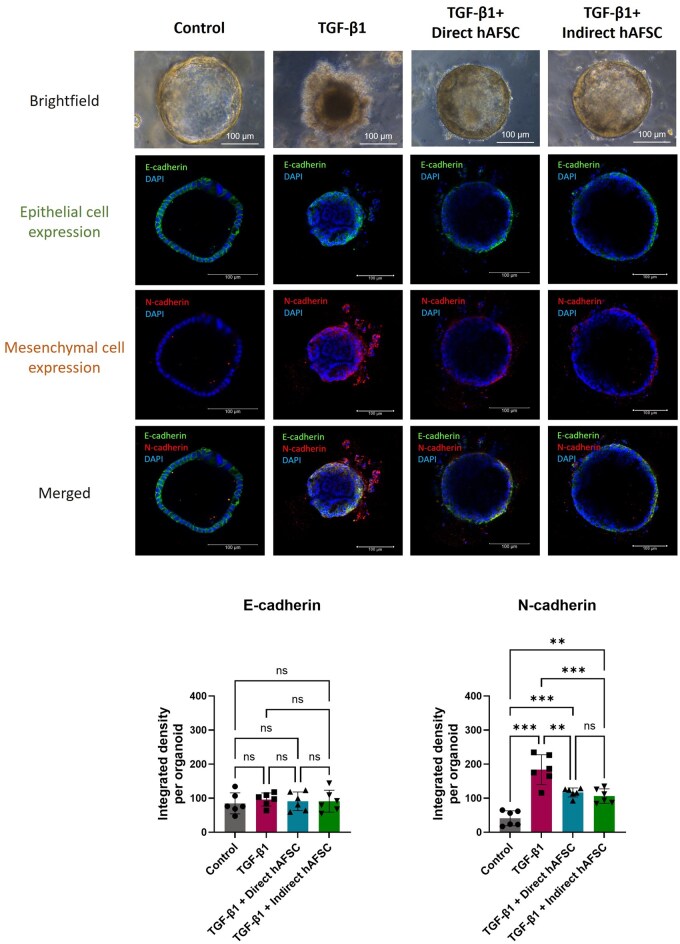
Protein expressions of biliary epithelial-mesenchymal transition (EMT) markers in liver ductal organoids after administration of human amniotic fluid stem cells (hAFSCs). Representative images of both bright field and immunofluorescent staining of liver ductal organoids. (Top) Immunofluorescent staining of epithelial cell marker E-cadherin (green), mesenchymal cell marker N-cadherin (red) and merged images in control group, TGF-β1 group, TGF-β1 with direct hAFSCs treatment group and TGF-β1 with indirect hAFSCs treatment group, and (bottom) the quantification of immunofluorescent expression. Nuclei were stained with DAPI (blue). Data are presented as mean ± SD. **P < 0.01, ***P < 0.001 (*N* = 6 per group).

Interestingly, after TGF-β1 administration, the morphology of liver ductal organoids exposed to hAFSCs in both direct and indirect co-culture systems maintained a spheroid shape with less outgrowth of epithelial cells compared to those not exposed to hAFSCs (Figure 4A). Analysis of mRNA expression in the organoids revealed that there were no changes in biliary epithelial cell markers, *Cytokeratin-19*, and *E-cadherin* (Figure 4B), and liver progenitor cell markers, *Alpha-fetoprotein* and *Cytokeratin-7*, in hAFSCs exposed groups compared to the not exposed group (Figure 4C). However, administration of hAFSCs in both direct and indirect co-culture systems could significantly decrease mRNA expression of mesenchymal cell markers including *N-cadherin*, *Vimentin* and *α-SMA* in liver ductal organoids receiving TGF-β1 (Figure 4D). These results were confirmed by double immunofluorescence staining of epithelial marker E-cadherin and mesenchymal marker N-cadherin. Although there was no change in protein expression of epithelial marker E-cadherin, both organoids directly and indirectly exposed to hAFSCs had a decreased protein expression of mesenchymal marker N-cadherin compared to the TGF-β1 group (Figure 5). These results demonstrated that hAFSCs could diminish the biliary EMT process in liver ductal organoids in both direct cell-cell interaction and paracrine manners.

### Expression of myofibroblast marker and collagen in liver ductal organoid undergoing biliary EMT were lowered by hAFSCs treatment in both direct and indirect co-culture systems

**Figure 6.**
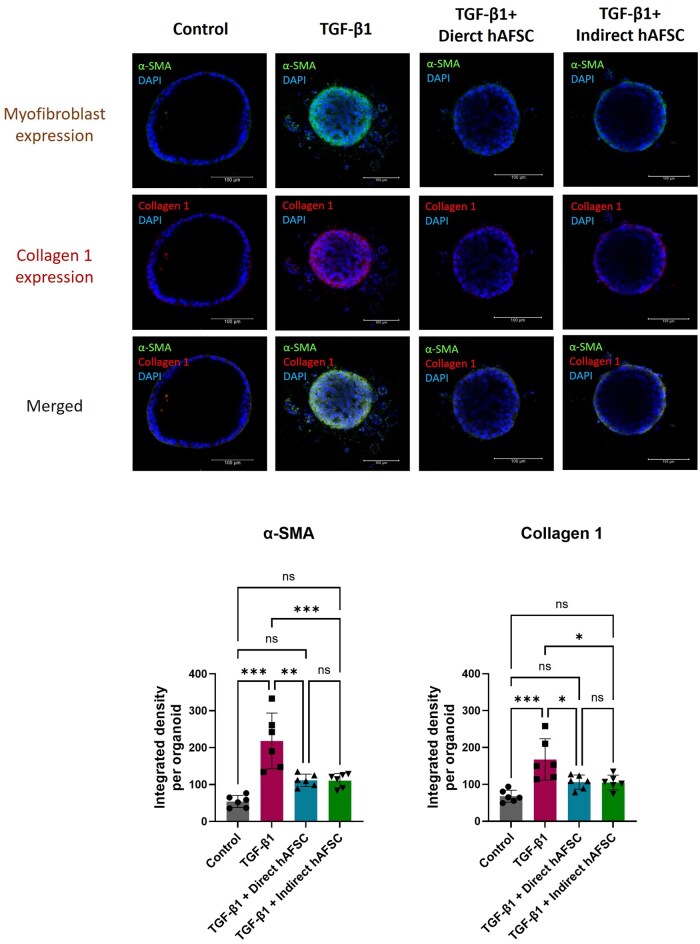
Correlation between protein expression of myofibroblast and collagen markers in liver ductal organoids after administration of human amniotic fluid stem cells (hAFSCs). Representative images of liver ductal organoids in double immunofluorescent staining of myofibroblast marker α-SMA (green) and Collagen 1 (red) in control group, TGF-β1 group, TGF-β1 with direct hAFSCs treatment group and TGF-β1 with indirect hAFSCs treatment group, and the quantification of immunofluorescent expression. Nuclei were stained with DAPI (blue). Data are presented as mean ± SD. * *P* < 0.05, **P < 0.01, ***P < 0.001.

mRNA expression analysis revealed that administration of hAFSCs in both direct and indirect co-culture systems could significantly decrease myofibroblast marker *α-SMA* and collagen marker *Col1a1* in liver ductal organoids receiving TGF-β1 (Figure 4D and E). To confirm the correlation between myofibroblast expression and collagen expression in the organoids, double immunofluorescent staining of α-SMA and Collagen1 was performed. We found that a decreased protein expression of α-SMA in both direct and indirect hAFSCs exposed groups was correlated with a decreased protein expression of Collagen1 in the organoids receiving TGF-β1 (Figure 6). These results demonstrated that hAFSCs treatment mitigated the transformation of biliary epithelial cell into myofibroblast, which resulted in reduced collagen expression in liver ductal organoids.

### hAFSCs downregulated Wnt signaling pathway in liver ductal organoids undergoing biliary EMT

**Figure 7.**
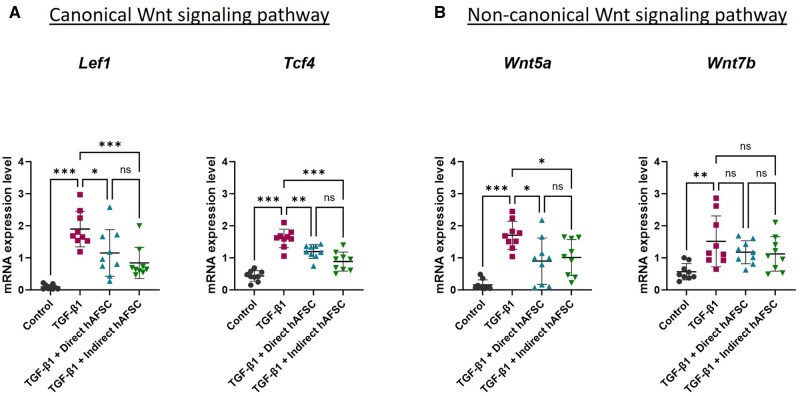
mRNA Expression of genes related to Wnt signaling pathways in liver ductal organoids. mRNA expression of genes related with (A) canonical Wnt signaling pathway including *Lef1* and *Tcf4*, and (B) non-canonical Wnt signaling pathway including *Wnt5a* and *Wnt7b* in control group, TGF-β1 group, TGF-β1 with direct hAFSCs treatment group, and TGF-β1 with indirect hAFSCs treatment group. The experiments were performed with 3 replicates (total *n* = 9 per group). Data are presented as mean ± SD. * *P* < 0.05, **P < 0.01, ***P < 0.001.

To explore the involvement of the Wnt signaling pathway in biliary EMT, we investigated mRNA expression of genes related to the canonical and non-canonical Wnt signaling pathways in liver ductal organoids. We found that the organoids receiving TGF-β1 increased mRNA expression of genes related to the canonical Wnt signaling pathway including *Lef1* and *Tcf4* (Figure 7A), as well as genes in the non-canonical Wnt pathway including *Wnt5a* and *Wnt7b* (Figure 7B). hAFSCs treatment in both direct and indirect co-culture systems can decrease the mRNA expressions of *Lef1*, *Tcf4* (Figure 7A), and *Wnt5a* (Figure 7B), but there was no significant change in *Wnt7b* expression (Figure 7B). Overall, these results suggest that the upregulation of Wnt signaling pathways in both canonical and non-canonical pathways is related to the biliary EMT process in liver ductal organoids. In addition, the downregulation of Wnt signaling by hAFSCs treatment, particularly in the modulation of Wnt5a in non-canonical Wnt signaling pathway, is associated with decreasing this EMT process.

## Discussion

Our study revealed that after administration of TGF-β1, the liver ductal organoids underwent biliary EMT, as demonstrated by increased expression of mesenchymal cell and myofibroblast markers. Moreover, the changing of biliary epithelial cells into myofibroblasts through EMT process was associated with an increased expression of collagen in the organoids. These findings support our hypothesis that the biliary EMT process is one of the mechanisms involved in fibrogenesis of the BA liver by transforming biliary epithelial cells into fibrogenic myofibroblasts, a population of collagen producing cells. Moreover, we discovered that hAFSCs could mitigate this EMT process by decreasing expression of myofibroblast in liver ductal organoids and altering their collagen production. Our further analysis also found that this anti-fibrotic effect of hAFSCs was related to the downregulation of genes related to Wnt signaling pathway.

Several studies in cholangiopathies including BA have revealed that RDCs in the liver undergo biliary EMT which is characterized by an increased expression of mesenchymal cell as well as myofibroblast markers in RDCs.^17^^,^^19^^,^^40^^,^^58–61^ However, the role of this biliary EMT process in liver fibrogenesis is still controversial. RDCs are the epithelial component of ductular reaction that express the biliary phenotype and are observed in the liver as bile duct proliferation.^62–64^ They are able to produce cytokines, chemokines, growth factors, and angiogenic factors. In addition, RDCs crosstalk with other components of ductular reaction including myofibroblasts, inflammatory cells, and endothelial cells.^65^ These RDCs can be derived from multiple cell sources including hepatic progenitor cells, pre-existing cholangiocytes, and periportal hepatocytes.^59^^,^^62^ In BA, there is evidence suggesting that RDCs are derived from the activation of Prom1-expressing hepatic progenitor cells.^66^ Therefore, we developed an *ex vivo* biliary EMT model using liver ductal organoids, which are derived from progenitor cells located in the intrahepatic bile duct, to mimic biliary EMT of RDCs.

The results of this study in the liver ductal organoid model are consistent with previous *in vitro* experimental studies investigating the mechanism of biliary EMT in human biliary epithelial cells, which demonstrated that TGF-β1 could induce EMT in biliary epithelial cells.^18^^,^^58^^,^^67^^,^^68^ We found that TGF-β1 induced the differentiation of bipotent liver progenitors into a bile duct progenitor and hepatocyte phenotypes, as indicated by the decreased expression of bipotent liver progenitor marker *Alpha-fetoprotein* and increased expression of bile duct progenitor markers *EpCAM*, *Cd133*, and *Cytokeratin-7*, as well as hepatocyte differentiation marker *Hnf1a*. Accordingly, our *ex vivo* biliary EMT model is suitable for studying the fibrogenesis process in biliary epithelial and progenitor cells. Ultimately, this research can be used to identify a potential target to inhibit this fibrogenesis process, as well as applied as a model for treatment screening. The limitation of this model includes the isolated investigation of EMT of bile duct without interaction with other cell types in the liver such as hepatic stellate cells and immune cells that are also involved in liver fibrogenesis. Therefore, further studies are needed to examine the biliary EMT process using assembloid which is an *in vitro* model that combines organoids with 2 or more types of cells in the liver. In addition, a prior study demonstrated that liver organoids derived from rhesus rotavirus-induced BA mice exhibited aberrant morphology and disturbed apical-basal organization, which were similar to the morphology of liver organoids derived from patients with BA. They had slow growth and produced spheres with multiple vacuoles which possibly associated with the alteration of beta-amyloid metabolism.^69^ Thus, additional study focusing on liver ductal organoids derived from experimental BA mice or from liver biopsies of BA patients may shed more light on the mechanisms of fibrogenesis in BA liver and could be used as a platform to evaluate the treatment.

Recent studies have revealed the properties of AFSCs in reducing fibrosis of organs in various experimental disease models.^50–55^ In a mouse model of carbon tetrachloride-induced liver fibrosis, it has been shown that AFSCs were able to engraft in the liver after administration of the stem cells via mesenteric vessels and improve liver fibrosis and hepatic function.^53^ However, the mechanism of AFSCs in reducing liver fibrosis still needs to be explored. In a mouse model of Alport syndrome with renal fibrosis, it has been shown that AFSCs could delay interstitial fibrosis and glomerular sclerosis by immunomodulation of macrophages.^54^ In a mouse model of unilateral ureteral obstruction, it has been shown that AFSCs could reduce interstitial fibrosis by regulation of growth factor and transcription factor including vascular endothelial growth factor, hypoxia inducible factor-1α, and TGF-β1.^55^ In addition, it has been shown that AFSCs could inhibit fibrotic alveolar and parenchymal remodeling in a mouse model of bleomycin-induced lung injury by modulating C-C motif chemokine ligand 2 (CCL2) through matrix metallopeptidase 2 (MMP2) mediated proteolytic cleavage.^51^ To investigate the anti-fibrotic effect of hAFSCs in biliary EMT of RDCs, the co-culture systems of hAFSCs with liver ductal organoids were applied in our biliary EMT model. Our results demonstrated that administration of hAFSCs as a treatment significantly attenuated the TGF-β1-induced biliary EMT and collagen production in liver ductal organoids (Figure 7). The results from our study are consistent with experimental studies in a mouse model of cutaneous skin wound which demonstrated the beneficial effect of AFSCs in decreasing scar formation. These effects function through paracrine fashion by decreasing the expression of myofibroblast (α‑SMA) and type I collagen in skin.^50^^,^^52^ Therefore, hAFSCs have the potential to prevent the progression of liver fibrosis in BA by decreasing the transformation of RDCs into myofibroblasts through biliary EMT process. In addition, by using the direct and indirect co-culture systems, we could confirm that the effect of hAFSCs in mitigating biliary EMT could happen not only in direct cell-cell interaction conditions but also through a paracrine manner. The paracrine signaling of hAFSCs includes extracellular vesicles (EVs), cytokines, bioactive factors, and microRNA. The size of hAFSCs EVs can range from 50 to 1000 nm^70^ which allows them to pass through the membrane and exert the therapeutic effect in our experiment. For future perspectives on the application of hAFSCs treatment, while the direct administration of stem cells may offer advantages in terms of cell-to-cell interactions and their abilities to regenerate and differentiate into other cell types, there is a risk of immunogenicity and tumorigenicity. Thus, the delivery of hAFSCs derivatives such as conditioned medium, EVs or microRNA is potentially beneficial to treat liver fibrosis in BA and to avoid the side effects of direct stem cell therapy. Further investigations using an experimental mouse model of BA are also essential to demonstrate the advantages of hAFSCs therapy in alleviating biliary EMT and liver fibrosis, as well as to assess its feasibility.

A major finding of our study is that the Wnt signaling is upregulated during the biliary EMT process in both canonical and noncanonical pathways, and modulation of this pathway by hAFSCs resulted in decreased expression of myofibroblast and collagen. Our findings are consistent with the studies of hepatobiliary and pancreatic cancers which revealed the involvement of the Wnt signaling pathway in promoting tumor invasion and metastasis by inducing EMT. Moreover, inhibition of the Wnt signaling pathway could decrease this EMT process.^71–74^ We also identified the correlation between Wnt5a, one of Wnt molecules, and the development of biliary EMT. Wnt5a is a secreted glycoprotein in the non-canonical members of Wnt signaling pathway. It plays a part in development, cell proliferation, and cell migration. In liver fibrosis, several studies have identified a functional role of Wnt5a in the fibrogenesis process.^75^^,^^76^ It has been found that, in human cirrhotic livers, Wnt5a expression was upregulated in myofibroblasts present in the fibrotic area. Moreover, incubation of human myofibroblasts with fibrogenic cytokine TGF-β resulted in a gradual increase of Wnt5a expression in myofibroblasts, which was also correlated with an increased collagen expression. In addition, the expression of mesenchymal marker and collagen was significantly reduced when Wnt5a was silenced in myofibroblasts treated with TGF-β^76^ indicating the role of Wnt5a in the development of liver fibrosis through the regulation of TGF-β-mediated myofibroblast differentiation and fibrogenesis. These results are consistent with our study, in which Wnt5a was upregulated in liver ductal organoids treated with TGF-β. The upregulation of Wnt5a in these organoids was associated with an increased expression of myofibroblasts, which occurred through the biliary EMT process. Thus, modulation of Wnt5a is a potential target to mitigate the biliary EMT process and decelerate progression of liver fibrosis.

## Conclusions

The biliary EMT of RDCs plays a role in BA liver fibrogenesis by transforming RDCs into myofibroblasts, which subsequently produce collagen. hAFSCs possess anti-fibrotic property that can mitigate biliary EMT process and reduce collagen production. Hence, the application of hAFSCs treatment in BA liver has potential to decelerate the progression of liver fibrosis. This study is relevant to the future implementation of precision medicine. Liver ductal organoids can be grown from liver biopsies and studied *ex vivo* to evaluate the development of fibrogenesis and to investigate whether a novel treatment such as hAFSCs administration could prevent biliary epithelial-mesenchymal transition.

## Supplementary Material

szaf052_Supplementary_Data
