# Supplementary material for: Anti-fibrotic effect of human amniotic fluid stem cells in biliary epithelial-mesenchymal transition of liver ductal organoid
Source: Stem Cells Transl Med. 2025 Nov 3;14(10):szaf052. doi: 10.1093/stcltm/szaf052 (PMC12582591; doi:10.1093/stcltm/szaf052)
Supplement: szaf052_Supplementary_Data [file szaf052_supplementary_data.docx]

**Supplementary Information**

**Anti-fibrotic effect of human amniotic fluid stem cells in biliary epithelial-mesenchymal transition of liver ductal organoid**

Sinobol Chusilp, MD^1,2^, Poramate Klanrit, MD^3^, Carol Lee, MSC^2^, Dorothy Lee, PhD^2^, Bo Li, PhD^2^, Felicia Balsamo, MSC^2^, Kanokrat Thaiwatcharamas, MD^1^, Patchareeporn Tanming, MD^1^, Dolrudee Aroonsaeng, MD^1^, Paisarn Vejchapipat, MD^4^, Agostino Pierro, MD^2^*

^1^Division of Pediatric Surgery, Department of Surgery, Faculty of Medicine, Khon Kaen University, Khon Kaen, 40002, Thailand

^2^Division of General and Thoracic Surgery, Translational Medicine Program, The Hospital for Sick Children, Toronto, ON, M5G 1X8, Canada

^3^Department of Systems Biosciences and Computational Medicine, Faculty of Medicine, Khon Kaen University, Khon Kaen, 40002, Thailand

^4^Division of Pediatric Surgery, Department of Surgery, Faculty of Medicine, Chulalongkorn University, Bangkok, 10330, Thailand


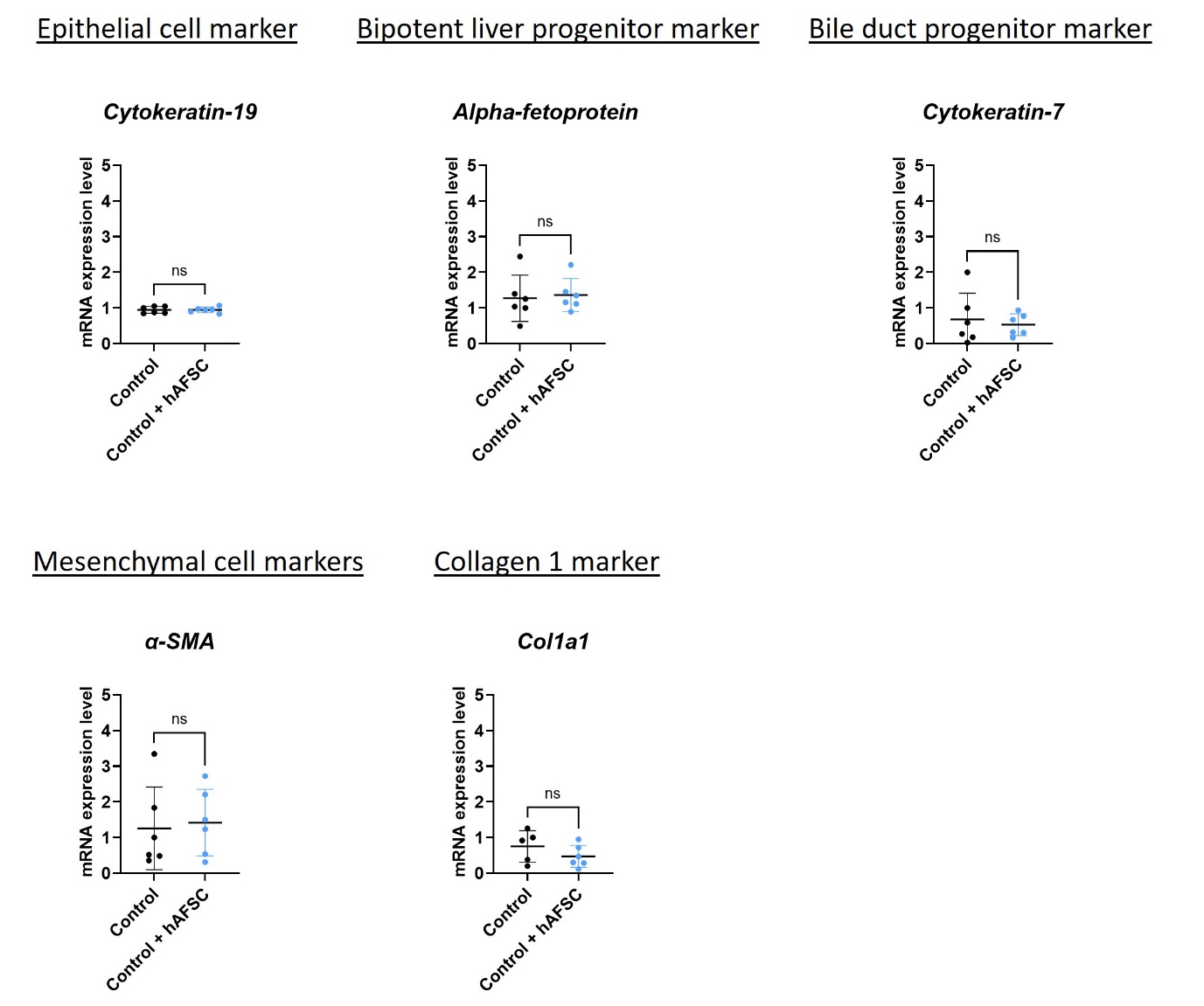


**Supplementary Figure. 1**

mRNA Expression of genes related to epithelial cell, bipotent liver progenitor, bile duct progenitor, mesenchymal cell, and collagen 1 markers in liver ductal organoids after at 48 hours without receiving TGF-β1 (normal condition). The experiments were performed with 2 replicates (total N = 6 per group). Data are presented as mean ± SD. ***P<0.001
